# Baseline Arterial CO_2_ Pressure Regulates Acute Intermittent Hypoxia-Induced Phrenic Long-Term Facilitation in Rats

**DOI:** 10.3389/fphys.2021.573385

**Published:** 2021-02-24

**Authors:** Raphael R. Perim, Mohamed El-Chami, Elisa J. Gonzalez-Rothi, Gordon S. Mitchell

**Affiliations:** Department of Physical Therapy, McKnight Brain Institute, Center for Respiratory Research and Rehabilitation, University of Florida, Gainesville, FL, United States

**Keywords:** acute intermittent hypoxia, phrenic long-term facilitation, respiratory plasticity, PaCO_2_, phrenic activity

## Abstract

Moderate acute intermittent hypoxia (mAIH) elicits a progressive increase in phrenic motor output lasting hours post-mAIH, a form of respiratory motor plasticity known as phrenic long-term facilitation (pLTF). mAIH-induced pLTF is initiated by activation of spinally-projecting raphe serotonergic neurons during hypoxia and subsequent serotonin release near phrenic motor neurons. Since raphe serotonergic neurons are also sensitive to pH and CO_2_, the prevailing arterial CO_2_ pressure (PaCO_2_) may modulate their activity (and serotonin release) during hypoxic episodes. Thus, we hypothesized that changes in background PaCO_2_ directly influence the magnitude of mAIH-induced pLTF. mAIH-induced pLTF was evaluated in anesthetized, vagotomized, paralyzed and ventilated rats, with end-tidal CO_2_ (i.e., a PaCO_2_ surrogate) maintained at: (1) ≤39 mmHg (hypocapnia); (2) ∼41 mmHg (normocapnia); or (3) ≥48 mmHg (hypercapnia) throughout experimental protocols. Although baseline phrenic nerve activity tended to be lower in hypocapnia, short-term hypoxic phrenic response, i.e., burst amplitude (Δ = 5.1 ± 1.1 μV) and frequency responses (Δ = 21 ± 4 bpm), was greater than in normocapnic (Δ = 3.6 ± 0.6 μV and 8 ± 4, respectively) or hypercapnic rats (Δ = 2.0 ± 0.6 μV and −2 ± 2, respectively), followed by a progressive increase in phrenic burst amplitude (i.e., pLTF) for at least 60 min post mAIH. pLTF in the hypocapnic group (Δ = 4.9 ± 0.6 μV) was significantly greater than in normocapnic (Δ = 2.8 ± 0.7 μV) or hypercapnic rats (Δ = 1.7 ± 0.4 μV). In contrast, although hypercapnic rats also exhibited significant pLTF, it was attenuated versus hypocapnic rats. When pLTF was expressed as percent change from maximal chemoreflex stimulation, all pairwise comparisons were found to be statistically significant (*p* < 0.05). We conclude that elevated PaCO_2_ undermines mAIH-induced pLTF in anesthetized rats. These findings contrast with well-documented effects of PaCO_2_ on ventilatory LTF in awake humans.

## Introduction

One of the most well-studied forms of respiratory motor plasticity is phrenic long-term facilitation (pLTF), characterized by a progressive increase in phrenic burst amplitude following moderate acute intermittent hypoxia (mAIH; Hayashi et al., 1993; Baker-Herman and Mitchell, 2002; Baker-Herman et al., 2004). During hypoxic episodes, carotid body neural network are activated, including brainstem neurons of the caudal, spinally-projecting raphe nuclei (Holtman et al., 1986; Pilowsky et al., 1990; Erickson and Millhorn, 1991, 1994; Morris et al., 1996). These raphe neurons release serotonin in the ventral spinal cord, including the phrenic motor nucleus (Kinkead et al., 2001), thereby activating spinal serotonin type 2 receptors that initiate an intracellular signaling cascade giving rise to pLTF (MacFarlane and Mitchell, 2009; Tadjalli and Mitchell, 2019).

Raphe serotonergic neurons are also activated directly by increased CO_2_ and/or decreased pH (Hodges and Richerson, 2010; Teran et al., 2014). On the other hand, hypercapnia amplifies carotid body hypoxic chemo-sensitivity (Lahiri and DeLaney, 1975; Kumar and Prabhakar, 2012), increasing synaptic inputs to raphe neurons. Thus, one might predict greater activation and serotonin-release from raphe neurons during hypoxia with a background of hypercapnia versus hypocapnia. Since both hypoxia (indirect) and CO_2_ (direct and indirect) modulate raphe serotonergic neuron activity, mAIH-induced pLTF expression may depend, at least in part, on the prevailing arterial CO_2_ pressure (PaCO_2_). The impact of background PaCO_2_ on pLTF has never been systematically investigated in anesthetized rats, although it’s impact on ventilatory LTF has been investigated extensively in humans (Harris et al., 2006; Syed et al., 2013; Tester et al., 2014).

mAIH-induced pLTF was first demonstrated in anesthetized, vagotomized, paralyzed and ventilated rats (Hayashi et al., 1993). Although not formally tested, the authors acknowledged that pLTF magnitude was greater when background PaCO_2_ was closer to the CO_2_ recruitment threshold, defined as the lowest end-tidal CO_2_ causing resumption of inspiratory phrenic bursts after hypocapnia-induced apnea. Since then, the existence of pLTF has been verified in many studies, typically with end-tidal CO_2_ regulated 2–3 mmHg above the recruitment threshold (Fuller et al., 2000; Baker-Herman and Mitchell, 2008). However, in several studies, anesthetized and spontaneously breathing rats failed to elicit diaphragm LTF (Janssen and Fregosi, 2000; Cao and Ling, 2010). These authors attributed the lack of diaphragm LTF either to: (1) hypercapnia inherent in spontaneously breathing, anesthetized rats (Janssen and Fregosi, 2000), and/or (2) the specific anesthetic or paralytic drugs used (Cao and Ling, 2010). Conversely, unanesthetized, spontaneously breathing rats exhibit robust ventilatory and/or diaphragm long-term facilitation (Olson et al., 2001; McGuire et al., 2008; Nakamura et al., 2010; Terada and Mitchell, 2011; Navarrete-Opazo and Mitchell, 2014a), demonstrating that normocapnic (versus hypercapnic) spontaneous breathing is compatible with LTF expression.

The main objective of the present study was to evaluate the effect of background PaCO_2_ on pLTF in the “standard” anesthetized and ventilated rat preparation. Contrary to expectations, we report that mAIH-induced pLTF is inversely correlated with baseline PaCO_2_ in rats, unlike humans (Harris et al., 2006; Syed et al., 2013; Tester et al., 2014). Possible factors contributing to CO_2_ interactions with pLTF, and differences between humans and rats are discussed. Our findings increase understanding of the diverse factors regulating pLTF expression. An understanding of these factors is essential to properly design future experiments, and for the translation of AIH-induced motor plasticity as a therapeutic modality to treat neuromuscular disorders that compromise respiratory and non-respiratory movements (Dale et al., 2014; Gonzalez-Rothi et al., 2015).

## Materials and Methods

### Animals

All experiments were approved by the University of Florida Institutional Animal Care and Use Committee (protocol #201408657). Adult male Sprague Dawley rats (329–415 g; 208A Colony, Envigo; IN, United States) were housed in pairs under standard conditions with 12:12-h light/dark cycle and free access to food and water. Sample sizes were estimated based on our extensive experience with this experimental preparation and knowledge of expected variance.

### Surgical Procedures

All surgical procedures have been previously described (Perim et al., 2018, 2019, 2020a,b). Rats were anesthetized in an acrylic chamber with 3% isoflurane in 3 L/min O_2_. They were weighed and transferred to a heated surgical table to regulate body temperature at 37.5 ± 1°C throughout experiments. Anesthesia was maintained with 3% isoflurane in 60% inspired O_2_ delivered through a nose cone. Additional inspired CO_2_ was added to keep end-tidal CO_2_ constant at target levels depending on the experimental group (see below).

Rats were tracheotomized with a 1 cm polyethylene catheter (I.D., 1.67 mm) for mechanical ventilation [0.007 × mass (g), ∼2.5 mL tidal volume; ∼70 breaths/min; VentElite small animal ventilator; Harvard Apparatus, Holliston, MA, United States]. Urethane (2.1 g/kg, 6 mL/hour) was administered through a tail vein catheter (24 Gauge; Surflash, Somerset, NJ, United States) while slowly reducing isoflurane until conversion was complete. Both vagus nerves were isolated and cut ∼1 cm caudal to thyroid cartilage. The right femoral artery was exposed and cannulated with a polyethylene catheter (I.D., 0.58 mm) to monitor blood pressure (Argon Pressure Transducer, DTXPlus, Plano, TX, United States) and sample arterial blood for blood gas analysis using heparinized capillary tubes (60 μL per blood sample; ABL 90 Flex, Radiometer, OH, United States).

Approximately 1 cm of the phrenic nerve was isolated near the brachial plexus, cut distally and partially de-sheathed to record electrical activity using suction electrodes. Signal was acquired at 25 kHz sampling frequency, amplified (1,000x), band-pass filtered (0.3–5 kHz) and digitalized using a differential amplifier (Model 1700, A-M Systems; Sequim, WA, United States) and an analog/digital converter (CED 1401; Cambridge Electronic Design, Cambridge, United Kingdom). Data were stored on a computer, rectified and smoothed with 50 ms time constant using Spike2 software (version 8.18; Cambridge Electronic Design; Cambridge, United Kingdom). Rats received the neuromuscular paralytic, pancuronium bromide (3 mg/kg, i.v., Sigma-Aldrich; Saint Louis, MO, United States), to eliminate spontaneous breathing efforts. Adequate anesthetic depth was confirmed by absence of withdrawal reflex or blood pressure response (i.e., after paralysis) to toe pinch. Fluids were administered intravenously (0.5–2.5 mL/h; 1:4 solution of 8.4% sodium bicarbonate mixed in standard lactated Ringer’s solution) to maintain acid-base balance.

### Experimental Design

Rats were randomly assigned to one of three groups: (1) Hypocapnia: end-tidal CO_2_ was maintained ≤ 39 mmHg during surgical procedures, and adjusted to ensure minimal rhythmic respiratory activity during baseline conditions as indicated by an unstable bursting pattern typically observed before a hypocapnia-induced apnea (Figure 3). End-tidal CO_2_ adjustments were made based on visual inspection of phrenic neurogram by an experienced investigator; (2) Normocapnia: end-tidal CO_2_ was maintained ∼41 mmHg throughout experiments. This level, based on previous studies, is ∼2 mmHg above the CO_2_ recruitment threshold (Perim et al., 2019, 2020a); (3) Hypercapnia: end-tidal CO_2_ was maintained ≥48 mmHg throughout experiments, corresponding to an average PaCO_2_ of ∼50 mmHg.

The mAIH protocol consisted of 3, 5-min hypoxic episodes (0.14 inspired O_2_ fraction) with 5-minute intervals (0.60 inspired O_2_ fraction). Targeted PaO_2_ during the last minute of hypoxic episodes was 35 to 55 mmHg; inspired O_2_ fraction was adjusted as necessary to remain within this range. Phrenic nerve activity was monitored for at least 60 min post-mAIH, while maintaining PaCO_2_, standard base excess and temperature at baseline values. At the end of experiments, all rat groups were exposed to maximal chemoreflex stimulation, consisting of hypoxia (0.10 inspired O_2_ fraction) combined with hypercapnia (0.07 inspired CO_2_ fraction) to assess maximal phrenic nerve activity.

### Data Analyses

Phrenic nerve activity was rectified and smoothed (0.05 s time constant) for off-line analyses. Peak phrenic burst amplitude and frequency were averaged over 1 min immediately before blood samples were taken at baseline, during the first hypoxic episode, and at 30 and 60 min post-mAIH. Data were analyzed using absolute values. We published a meta-analysis comparing absolute values of integrated phrenic nerve activity across groups and conclude that it is highly repeatable when adequate precautions are taken (Nichols and Mitchell, 2016), including adequate experimenter experience/skill, consistent electrode properties and recording setup. Experiments were only considered in the analysis if: (1) PaCO_2_ ± 1.5 mmHg and mean arterial pressure ± 30 mmHg relative to baseline; (2) PaO_2_ was > 150 mmHg during baseline and post-mAIH, and within the predefined range during hypoxia; and (3) the phrenic response to maximal chemoreceptor stimulation was greater than during hypoxic episodes. In total, 1 rat from normocapnic and 1 rat from the hypercapnic group were not considered for analysis based on the latter exclusion criteria. Normal distribution of residual errors was confirmed by visual inspection of histograms and normal probability plots. A one-way ANOVA was used to compare mean response among groups. Pairwise comparisons were carried out when appropriate using Fisher’s Least Significant Difference *post hoc* test. Values are expressed as mean ± 1 standard error of the mean. An alpha-level of 0.05 was used to assess statistical significance for all comparisons. All analyses were carried out using R Version 3.4.2 (R Core Team, Vienna, Austria).

## Results

**FIGURE 1:**
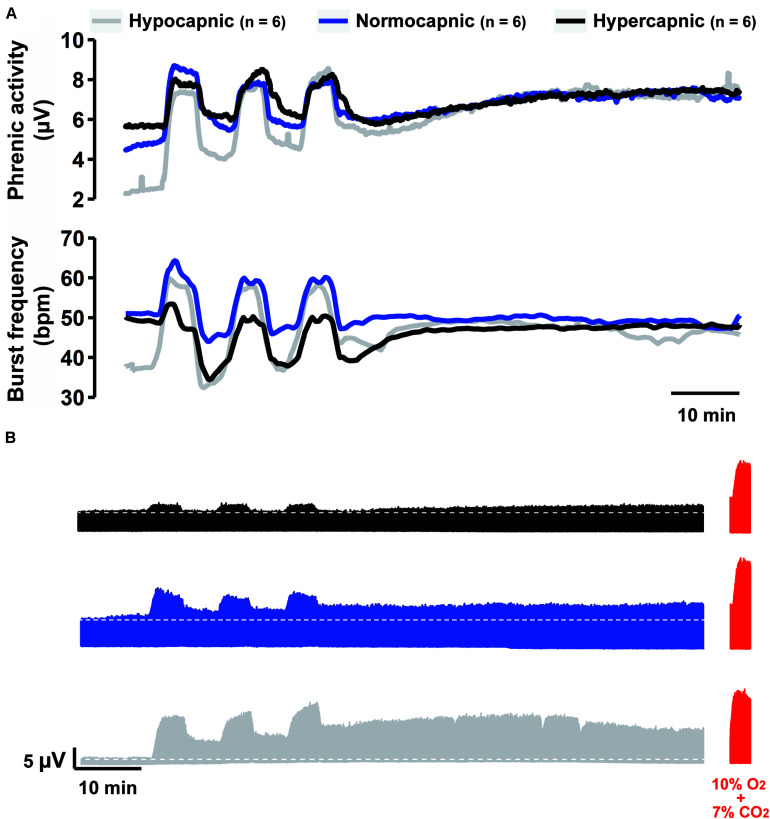
Overall summary of our findings. **(A)** Average traces of phrenic activity (top) and burst frequency (bottom) throughout the mAIH protocol from rat groups kept under constant hypocapnia (gray line), normocapnia (blue line) or hypercapnia (black line). **(B)** Group-representative phrenic neurograms during mAIH protocol. At the end of each experiment (shown in red), maximal phrenic output was assessed with 10% oxygen plus 7% CO_2_ (i.e., maximal chemoreflex stimulation, MCS).

Figure 1A shows average phrenic burst amplitude and frequency throughout the mAIH protocol. This form of data presentation provides a thorough examination of the outcome, which often leads to identification of group-specific trends not considered in *a priori* defined analyses. For example: (1) all groups reached similar absolute phrenic burst amplitudes 60 min post-mAIH, despite different background respiratory drive (i.e., as indicated by PaCO_2_); (2) the hypercapnic group had a blunted burst frequency response during hypoxia, which remained below baseline levels following mAIH. This burst frequency response pattern during mAIH was not expected based on previous studies from our laboratory (Fuller et al., 2000; Baker-Herman and Mitchell, 2008) and was clearly not observed in hypocapnic or normocapnic groups. A correlation between frequency LTF and baseline burst frequency has been noted before (Baker-Herman and Mitchell, 2008). Figure 1B shows group-representative phrenic neurograms, and Table 1 describes blood gases and mean arterial pressure throughout the protocol.

**TABLE 1:** Stability of blood samples and mean arterial pressure (MAP) among experimental groups throughout the moderate acute intermittent hypoxia protocol (mAIH).

	Experimental groups		
	
	Hypocapnic	Normocapnic	Hypercapnic
**PaO_2_, mmHg**	(*n* = 6)	(*n* = 6)	(*n* = 6)
Baseline	313 ± 10	287 ± 16	269 ± 15
Hx	43.9 ± 2*	41.2 ± 1*	42.6 ± 2*
30	256 ± 18	280 ± 12	236 ± 16
60	282 ± 14	276 ± 12	263 ± 12
**PaCO_2_, mmHg**			
Baseline	41.9 ± 1.1	43.9 ± 0.7	54.1 ± 0.5
Hx	41.0 ± 1.4	44.3 ± 0.7	52.8 ± 0.8
30	42.1 ± 1.5	45.4 ± 0.9	51.9 ± 1.5
60	42.4 ± 1.2	44.6 ± 0.9	54.6 ± 0.9
**sBE, mmol/L**			
Baseline	2.2 ± 1.1	2.4 ± 0.7	2.2 ± 0.4
Hx	−0.2 ± 0.6	0.7 ± 0.9	0.3 ± 0.7
30	−0.3 ± 1.1	0.6 ± 1.3	1.0 ± 0.8
60	0.8 ± 1.2	0.3 ± 0.8	2.5 ± 0.6
**Ph**			
Baseline	7.41 ± 0.01	7.40 ± 0.01	7.33 ± 0.00
Hx	7.39 ± 0.01	7.38 ± 0.01	7.31 ± 0.00
30	7.37 ± 0.01	7.36 ± 0.02	7.33 ± 0.01
60	7.39 ± 0.01	7.37 ± 0.01	7.33 ± 0.01
**ctHb, g/dL**			
Baseline	16.4 ± 0.2	16.6 ± 0.2	16.0 ± 0.1
Hx	15.8 ± 0.2	16.3 ± 0.0	15.8 ± 0.2
30	15.6 ± 0.4	16.3 ± 0.2	15.1 ± 0.4
60	15.6 ± 0.3	15.7 ± 0.2	15.4 ± 0.3
**MAP, mmHg**			
Baseline	120 ± 7	144 ± 3	133 ± 5
Hx	83 ± 9*	104 ± 6*	111 ± 13*
30	110 ± 6	122 ± 4	126 ± 5
60	117 ± 7	124 ± 4	136 ± 6

*Asterisks indicate significant differences in PaO_2_ and MAP relative to baseline values (*p* < 0.05). ANOVA indicated that only group main effect was significant (*p* < 0.001); pairwise comparisons of group main effect show that PaCO_2_ is lowest in the hypocapnic followed by normocapnic and then the hypercapnic rats.*

**FIGURE 2:**
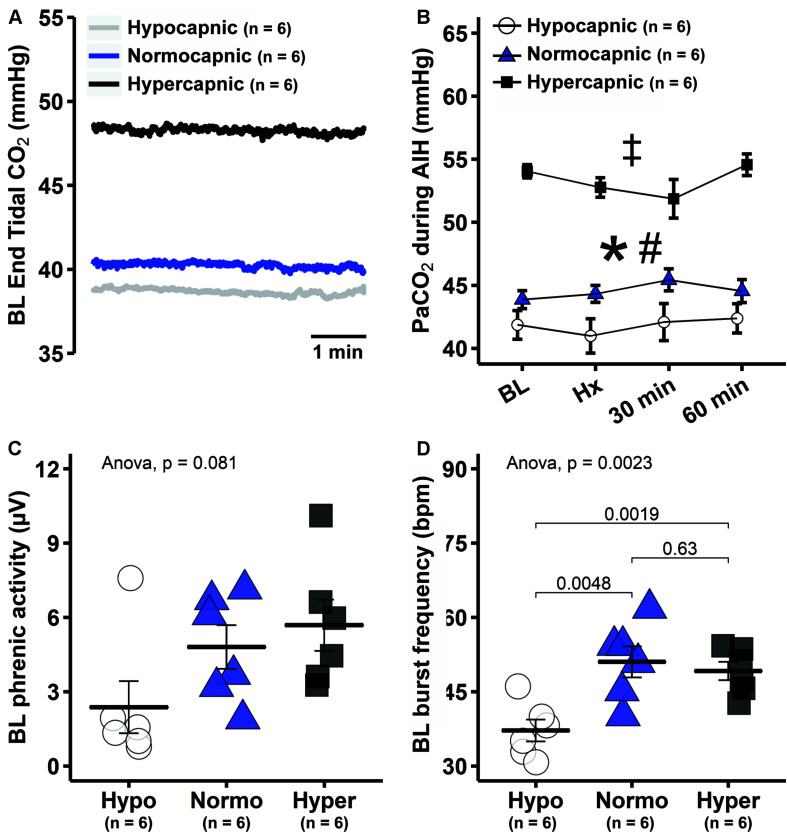
Reduced background PaCO_2_ in hypocapnic rats led to lower baseline respiratory activity. **(A)** Group average end-tidal CO_2_ traces during baseline. **(B)** Average PaCO_2_ at specific time points during mAIH protocol in hypocapnic, normocapnic and hypercapnic groups. ANOVA indicated that only group main effect was significant (*p* < 0.001); pairwise comparisons of group main effect show that PaCO_2_ is lowest in the hypocapnic followed by normocapnic and then the hypercapnic rats. *, significant differences between hypocapnic and normocapnic group; #, significant differences between normocapnic and hypercapnic group; ‡, significant differences between hypocapnic and hypercapnic group. **(C)** Group average baseline phrenic burst amplitude. **(D)** Group average phrenic burst frequency. Data are presented as mean ± standard error of the mean.

Group average end-tidal CO_2_ during baseline is presented in Figure 2A. After baseline, the end-tidal CO_2_ trace is omitted since it was used only as a guide to help maintain isocapnia. Typically, end-tidal CO_2_ was ∼2 mmHg below baseline PaCO_2_. During and post mAIH, isocapnia was determined by PaCO_2_ analysis (Figure 2B). There was a significant group effect of PaCO_2_ (*p* < 0.001), but no effect of time (*p* = 0.43) or time × group interaction (*p* = 0.35) detected by mixed two-way ANOVA. Pairwise comparisons of group main effect showed that PaCO_2_ in the hypocapnic group was lower versus normocapnic (*p* = 0.0218) or hypercapnic groups (*p* < 0.001); and lower in the normocapnic versus hypercapnic group (*p* < 0.001). Because PaCO_2_ is a major determinant of respiratory depth and rate, phrenic burst amplitude and frequency progressively increase with PaCO_2_ as expected (Figures 2C,D). Although one-way ANOVA did not reach statistical significance for baseline phrenic burst amplitude among groups (*p* = 0.08), this result was affected by an outlier in the hypocapnic group, confirmed by a two-tailed Grubb’s test (*p* = 0.0014). When this outlier was no longer considered in the analysis, the ANOVA was highly significant (*p* = 0.0078). Then, pairwise comparisons showed that phrenic burst amplitude in the hypocapnic group was lower than in normocapnic (*p* = 0.0066) and hypercapnic groups (*p* = 0.0046). Similarly, baseline frequency was significantly reduced in the hypocapnic versus normocapnic (*p* = 0.0048) and hypercapnic groups (*p* = 0.0019).

**FIGURE 3:**
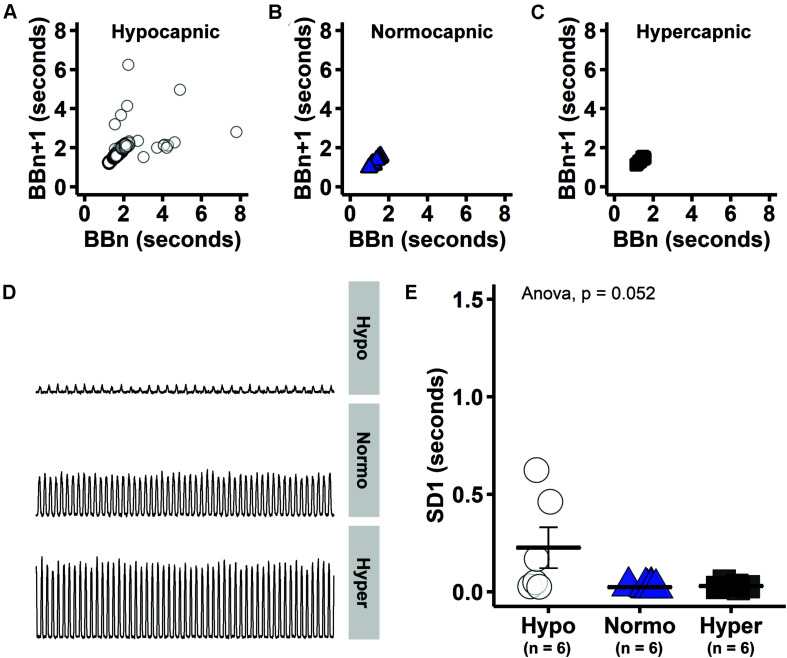
Consecutive phrenic burst intervals tended to be more variable in hypocapnic group, consistent with unstable breathing that is expected near CO_2_ apneic threshold. **(A–C)** Consecutive phrenic burst intervals (i.e., BBn versus BBn + 1) in hypocapnic, normocapnic and hypercapnic group, respectively. **(D)** Group-representative baseline phrenic activity. **(E)** Measure of Poincaré length that indicates long-term variability (SD1).

Poincaré plots indicate that there is considerable variability between consecutive phrenic burst intervals (i.e., BBn versus BBn + 1) in the hypocapnic group (Figure 3A). On the other hand, normocapnic and hypercapnic groups presented more symmetrical phrenic burst intervals during baseline (Figures 3B,C). Visual inspection of group-representative baseline phrenic activity also indicates unstable pattern with low PaCO_2_ (Figure 3D). The oscillation between consecutive phrenic burst intervals was quantified by SD1 measure of short-term variability (Poincaré cloud width). Group average SD1 tended to be higher in hypocapnic rats (Figure 3E), but did not reach statistical significance in one-way ANOVA (*p* = 0.052). Increased variability in consecutive burst intervals during hypocapnia provide evidence of unstable breathing at low respiratory drive. PaCO_2_ was just sufficient to maintain rhythmic bursting in the hypocapnic group.

**FIGURE 4:**
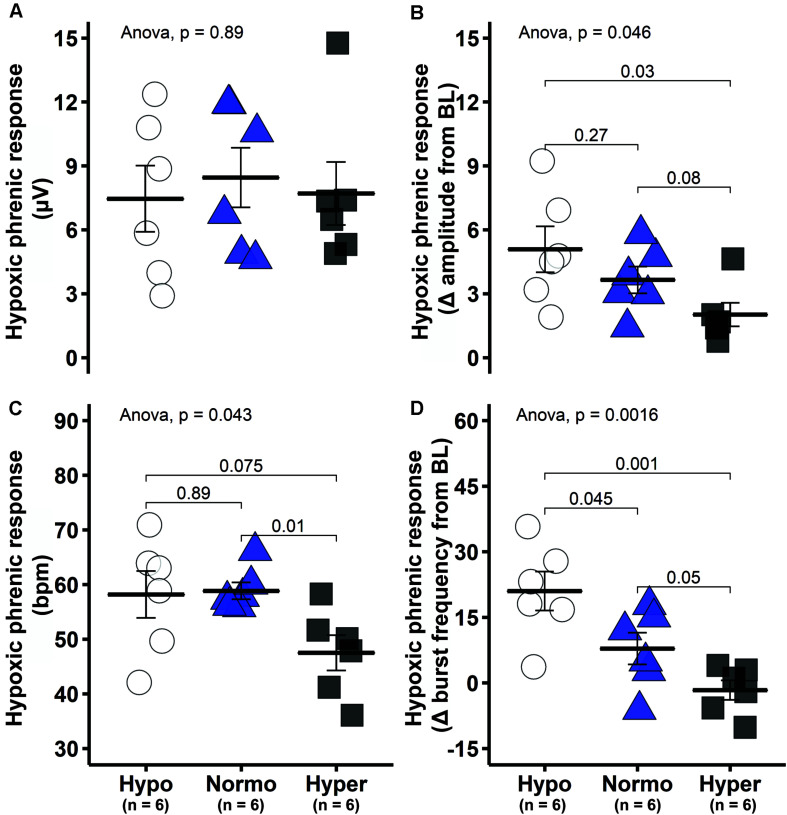
Short-term hypoxic phrenic response was enhanced in the hypocapnic group. **(A)** Group average short-term hypoxic phrenic response in absolute burst amplitude values. **(B)** Group average short-term hypoxic phrenic response as delta burst amplitude from baseline. **(C)** Group average short-term hypoxic phrenic response in absolute burst frequency values. **(D)** Group average short-term hypoxic phrenic response as delta burst frequency from baseline. Data are presented as mean ± standard error of the mean.

The short-term hypoxic phrenic response, expressed as absolute phrenic burst amplitude (μV) was similar among groups (Figure 4A), despite different baseline values (Figures 1A, 2D). A better approach under this condition is to assess hypoxic phrenic response as a change in phrenic activity from baseline (Δ from baseline), since it accounts for differences in background activity. The hypoxic phrenic response expressed this way was higher in hypocapnic versus hypercapnic rats (*p* = 0.03; Figure 4B). Burst frequency during hypoxic episodes showed a similar pattern. The hypocapnic and normocapnic groups tended to present higher hypoxic frequency responses to hypoxia versus hypercapnia when expressed in absolute values (*p* = 0.075 and *p* = 0.01, respectively), although only normocapnic versus hypercapnic comparison was significant (Figure 4C). Analyzed as a change from baseline, the frequency response was higher in hypocapnia versus normocapnia (*p* = 0.045) and hypercapnia (*p* = 0.001, Figure 4D).

**FIGURE 5:**
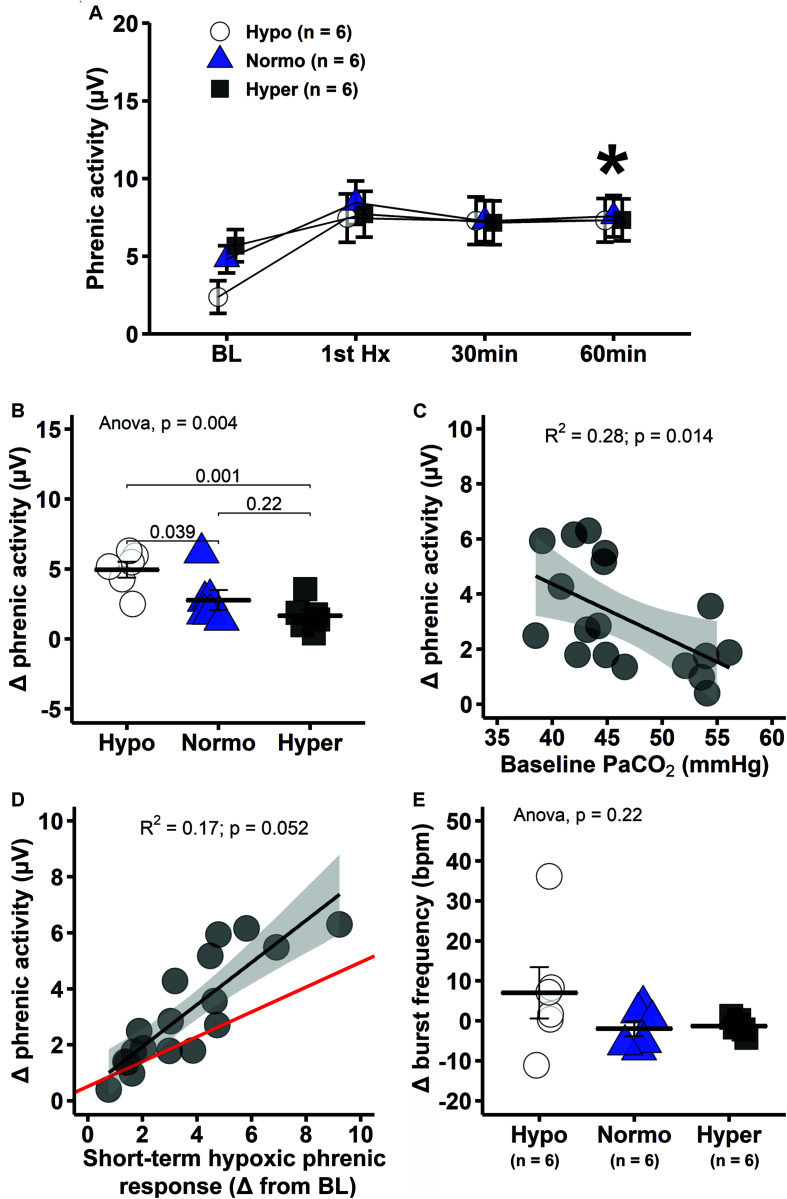
Hypocapnic rats presented the greatest pLTF, which decreases progressively with increasing PaCO_2_. **(A)** Average phrenic burst amplitude at specific times during mAIH protocol. Asterisk indicates significant differences at 60 min post-mAIH compared to baseline within hypocapnic, normocapnic and hypercapnic group (mixed ANOVA; *p* = 0.002514 for group × time interaction). **(B)** Average change in phrenic burst amplitude from baseline to 60 min post-mAIH. **(C)** Linear regression between baseline PaCO_2_ and change in phrenic burst amplitude from baseline to 60 min post-mAIH. **(D)** Linear regression between short-term hypoxic phrenic response and change in phrenic burst amplitude from baseline to 60 min post-mAIH. Bayesian regression line (red) describes the association between short-term hypoxic phrenic response and pLTF magnitude, taking into account prior data from our laboratory (Fuller et al., 2000; Baker-Herman and Mitchell, 2008). The 95% confidence interval of the regression slope was 0.4 and 0.5. **(E)** Average change in phrenic burst frequency from baseline to 60 min post-mAIH. Data are presented as mean ± standard error of the mean.

Mixed two-way ANOVA with phrenic burst amplitude as dependent variable shows significant interaction between group and time (*p* = 0.0025). Pairwise comparisons within groups indicate that phrenic burst amplitude was elevated at 60 min post-mAIH in all groups relative to baseline, demonstrating development of pLTF (*p* < 0.05; Figures 1A, 5A). However, pLTF magnitude varied substantially among groups. In hypocapnia, the change in phrenic burst amplitude versus baseline at 60 min post-mAIH was significantly higher than in normocapnia (*p* = 0.039) or hypercapnia (*p* = 0.001; Figure 5B). Linear regression analysis showed a significant negative correlation between baseline PaCO_2_ and pLTF (*R*^2^ = 0.28, *p* = 0.014; Figure 5C). The correlation between the hypoxic phrenic response and pLTF 60 min post-mAIH did not reach statistical significance (*R*^2^ = 0.17, *p* = 0.052; Figure 5D). The association between hypoxic phrenic response and pLTF magnitude has been assessed in two meta-analysis from our laboratory and a positive correlation was found in both studies. Thus, we used Bayesian inference (rjags package) to account for relevant prior information when interpreting p-values (Fuller et al., 2000; Baker-Herman and Mitchell, 2008), in accordance with recent statistical guidelines (Kennedy-Shaffer, 2019; Wasserstein et al., 2019). The 95% confidence intervals (0.4 and 0.5) of the Bayesian regression line slope indicated a positive correlation between hypoxic phrenic response and pLTF magnitude.

Although pLTF is often reported as percent change from baseline, we intentionally omitted data normalized in this way since baseline phrenic burst amplitude was different among groups; thus, results based on normalized data would be misleading, reflecting baseline versus pLTF changes. Average frequency change at 60 min post-mAIH tended to be higher in hypocapnia, but this small difference was not statistically significant (*p* = 0.22; Figure 5E).

**FIGURE 6:**
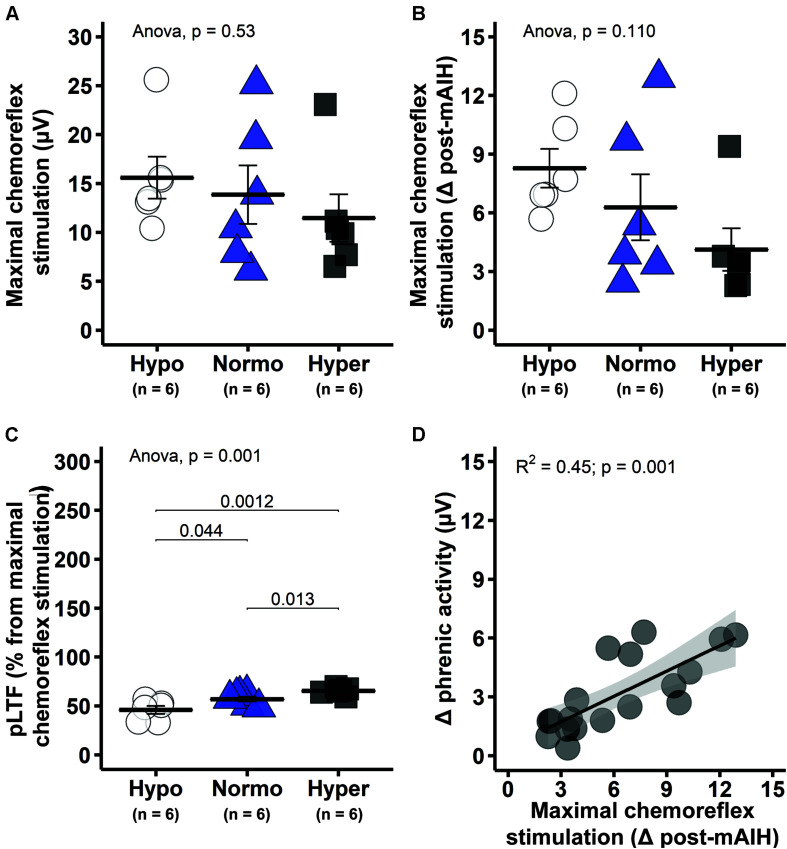
Maximal chemoreflex stimulation (MCS) tended to be greater in hypocapnic rats, suggesting enhanced capacity following mAIH. **(A)** Group average phrenic response to maximal chemoreflex stimulation in absolute burst amplitude values. **(B)** Average phrenic response to maximal chemoreflex stimulation as delta burst amplitude from 60 min post-mAIH (pre-stimulus level). **(C)** Average magnitude of phrenic long-term facilitation (pLTF) as percent from maximal chemoreflex stimulation. **(D)** Linear regression between phrenic response to maximal chemoreflex stimulation [data from panel **(B)**] and delta phrenic burst amplitude from baseline to 60 min post-mAIH. Data are presented as mean ± standard error of the mean.

Phrenic response to maximal chemoreflex stimulation showed a consistent pattern across analytical approaches. There were no significant differences in phrenic burst amplitude (absolute values) between groups (one-way ANOVA, *p* = 0.53; Figure 6A). Although significant differences were not found when phrenic response to maximal chemoreflex stimulation was expressed as a change from 60 min post-mAIH (one-way ANOVA, *p* = 0.110; Figure 6B), trends became significant when expressed as percent change in phrenic activity versus pre-stimulation values (one-way ANOVA, *p* = 0.006; data not shown), with lowest values found in the hypercapnic group. Interestingly, pLTF magnitude quantified as percent change from maximal chemoreflex stimulation was lower in hypocapnic rats, followed by normocapnic and then hypercapnic group (*p* < 0.05; Figure 6C), demonstrating that our results are not an artifact of phrenic neurogram saturation or changes in baseline due to the different background PaCO_2_ levels. There was a significant correlation between phrenic activity during maximal chemoreflex activation and pLTF (*R*^2^ = 0.45, *p* = 0.001; Figure 6D).

## Discussion

Contrary to expectations, mAIH-induced pLTF is greater with background PaCO_2_ levels near the CO_2_ apneic threshold; pLTF decreases as background PaCO_2_ is increased in anesthetized rats. Short-term hypoxic phrenic response (both burst amplitude and frequency) was also greater in hypocapnia, which confirms previously published meta-analyses of large data sets, suggesting that short-term hypoxic phrenic response is a strong predictor of pLTF magnitude (Fuller et al., 2000; Baker-Herman and Mitchell, 2008). Since this response was enhanced with lower background PaCO_2_ in the present study, it may help explain increased pLTF magnitude during hypocapnia.

Raphe serotonergic neuron activation during mAIH triggers serotonin release into the phrenic motor nucleus (Kinkead et al., 2001), initiating a serotonin receptor type 2-dependent signaling cascade to pLTF (Tadjalli and Mitchell, 2019). Thus, one key determinant of pLTF is the extent of AIH-induced raphe serotonergic neuron activation. Since conditions that mitigate serotonergic neuron activity during hypoxia are expected to undermine pLTF, the present results were unexpected.

Raphe serotonergic neurons are CO_2_ sensitive, responding rapidly to increased CO_2_ or decreased pH (Hodges and Richerson, 2010; Teran et al., 2014). Since raphe neurons decrease their activity with hypocapnia (Nuding et al., 2015), they may have a greater relative increase in (but lower level of) activity in response to hypoxia at low background PaCO_2_ levels. Further, hypoxia and CO_2_ are synergistic in their actions on carotid body chemoreceptors (Lahiri and DeLaney, 1975), predicting greater absolute raphe neuron activity and serotonin release in relative hypercapnia, although the physiological impact of serotonin release during hypoxia may be greatest at low background PaCO_2_ levels. Several lines of evidence suggest that episodic (not continuous) serotonin release is a more relevant stimulus to synaptic plasticity (Carew et al., 1972; Clark and Kandel, 1993; Emptage and Carew, 1993; Baker et al., 2001; MacFarlane and Mitchell, 2009), and that low-dose serotonin is a more potent stimulus to phrenic motor plasticity (MacFarlane and Mitchell, 2009). Thus, episodic raphe serotonergic neuron activation in a low (hypocapnic) versus high (hypercapnic) range may elicit greater mAIH-induced pLTF based on the dose-response relationship between phrenic motor facilitation and serotonin release. However, it is unknown whether serotonergic raphe neuron responses to mAIH vary with the prevailing brain tissue CO_2_ level. From a very different perspective, the impact of background PaCO_2_ on mAIH-induced pLTF may arise from the integrative properties of phrenic motor neurons. Accumulating evidence suggests that mAIH elicits plasticity within phrenic motor neurons *per se* (Devinney et al., 2015; Dale et al., 2017). Whether the hypocapnia increases mAIH-induced pLTF magnitude *via* differential regulation of raphe serotonergic neurons was not tested in the present study. This possibility deserves further investigation.

In some experimental preparations, a negative interaction between central (hypercapnia) and peripheral chemoreceptors (hypoxia) has been reported (Day and Wilson, 2008, 2009). For example, phrenic responses to isolated carotid body hypoxia are enhanced when the brainstem is independently perfused with 25 versus 50 mmHg PaCO_2_ (Day and Wilson, 2007). If this finding mirrors increased short-term hypoxic phrenic response and raphe neuron activation in hypocapnic rats, it is consistent with augmented short-term hypoxic phrenic response and pLTF in hypocapnic rats, as found here. An augmented hypoxic response of brainstem respiratory neurons in hypocapnia might produce greater serotonergic neuron activation and pLTF *via* indirect projections from brainstem ventral respiratory group neurons to midline raphe (Morris et al., 1996).

In a previous report, baseline PaCO_2_ was suggested (not demonstrated) to impact pLTF expression (Hayashi et al., 1993). Interestingly, diaphragm LTF was not observed when mAIH was applied in anesthetized, spontaneously breathing rats (Janssen and Fregosi, 2000). These authors suggested that this absence of diaphragm LTF was likely due to the presence of hypercapnia characteristic of anesthetized, spontaneously breathing rats. Accordingly, subsequent studies demonstrating robust AIH-induced ventilatory and/or diaphragm LTF in unanesthetized, spontaneously breathing and normocapnic rats (Olson et al., 2001; McGuire et al., 2008; Nakamura et al., 2010; Terada and Mitchell, 2011; Navarrete-Opazo and Mitchell, 2014a) are consistent with the idea that baseline PaCO_2_, and not spontaneous breathing *per se* suppressed diaphragm LTF. We acknowledge that other factors may have been involved, such as the specific anesthetic used. For example, Cao and Ling (2010) reported genioglossus LTF in spontaneously breathing rats anesthetized with a chloralose/urethane mixture, but not urethane alone.

Although mAIH-induced phrenic motor plasticity is primarily expressed as an increase in phrenic burst amplitude in rats (Hayashi et al., 1993; Baker-Herman et al., 2004; Baker-Herman and Mitchell, 2008; Nakamura et al., 2010; Terada and Mitchell, 2011; Perim and Mitchell, 2019), small increases in post-mAIH burst frequency have been reported (Baker-Herman and Mitchell, 2008). In a meta-analysis, baseline phrenic burst frequency was inversely correlated with the magnitude (and sign) of frequency LTF; frequency changes within hypoxic episodes, and pLTF magnitude are also positively correlated with frequency LTF. Nevertheless, since frequency LTF is less than 20% of pLTF amplitude in anesthetized rats (Baker-Herman and Mitchell, 2008), it is not surprising that only a non-significant trend toward frequency LTF occurs in hypocapnic rats.

Unlike most prior studies from our group using this same experimental preparation, the CO_2_ apneic/recruitment threshold was not determined in the present study. The CO_2_ recruitment threshold is an important reference condition adopted in most studies from our laboratory to normalize respiratory activity among rats within and across studies. We intentionally omitted this procedure here since our main goal was to investigate background CO_2_ effects on pLTF and, therefore, we hoped to minimize influences from other variables. For example, prolonged or repetitive central neural apnea is sufficient to trigger a distinct, interacting form of respiratory motor plasticity known as inactivity-induced phrenic motor facilitation which, although phenotypically similar to pLTF, occurs *via* distinct mechanisms (Strey et al., 2012; Baertsch and Baker-Herman, 2015; Baertsch and Baker, 2017). Brief repetitive apneas, leading to inactivity-induced phrenic motor facilitation, constrain mAIH-induced pLTF in rats (Fields et al., 2019). Although the apnea protocols most often used to elicit inactivity-induced phrenic motor facilitation are different in pattern and duration from our apnea/recruitment threshold determination (Strey et al., 2012; Baertsch and Baker-Herman, 2015; Baertsch and Baker, 2017), the impact of apneic/recruitment threshold determination on pLTF magnitude is simply not known.

Multiple brain regions have chemoreceptor neurons, including the retrotrapezoid nucleus (Mulkey et al., 2004), nucleus tractus solitaries (Dean et al., 1990), locus coeruleus (Pineda and Aghajanian, 1997) and hypothalamus (Dillon and Waldrop, 1992). Phox2b-expressing retrotrapezoid neurons and raphe serotonergic neurons project throughout the respiratory network and contribute to maximal chemoreflex stimulated phrenic responses (Severson et al., 2003; Mulkey et al., 2004; Corcoran et al., 2013). Although retrotrapezoid neurons have intrinsic CO_2_/pH sensitivity, part of their chemosensitivity appears to result from serotonergic neuronal inputs (Wu et al., 2019). Thus, enhanced phrenic responses to maximal chemoreflex activation in hypocapnia may be explained by reduced pre-stimulus raphe neuron activity (i.e., low PaCO_2_), leading to greater relative activity changes during maximal chemoreflex activation.

In awake humans, AIH-induced ventilatory LTF occurs in healthy individuals and in people with obstructive sleep apnea or spinal cord injury, but only if baseline PaCO_2_ is elevated by ∼2 mmHg (Harris et al., 2006; Lee et al., 2009; Gerst et al., 2011; Tester et al., 2014). The need for supplemental CO_2_ in humans is in direct contrast with our results in anesthetized paralyzed and ventilated rats. It is not known if this discrepancy is due to species or technical issues. However, a negative correlation between baseline ventilation and ventilatory long-term facilitation has been reported (Gerst et al., 2011), suggesting a negative CO_2_ influence at more extreme levels. Ventilatory LTF is often expressed in humans without supplemental CO_2_ during sleep, where PaCO_2_ is elevated by loss of the “wakefulness drive” (Babcock and Badr, 1998; Shkoukani et al., 2002; Babcock et al., 2003; Mateika and Sandhu, 2011).

Here, we demonstrate that background PaCO_2_ level is a key factor that can influence the capacity to elicit mAIH-induced pLTF expression. At PaCO_2_ levels barely sufficient to maintain rhythmic phrenic activity, both the short-term hypoxic phrenic response and pLTF are enhanced. These findings increase our understanding of factors regulating pLTF. Greater understanding of respiratory motor plasticity is relevant in at least 2 translationally relevant contexts: (1) it increases our understanding about non-respiratory motor systems and their response to AIH (Lovett-Barr et al., 2012; Prosser-Loose et al., 2015), and (2) it guides/refines our ability to harness repetitive mAIH as a therapeutic modality to treat breathing and other movement disorders during devastating traumatic, ischemic, infectious and/or neurodegenerative disorders that compromise movements, including breathing (Mitchell, 2007; Dale et al., 2014; Navarrete-Opazo and Mitchell, 2014b; Gonzalez-Rothi et al., 2015).

## Data Availability Statement

The original contributions presented in the study are included in the article/supplementary material, further inquiries can be directed to the corresponding author/s.

## Ethics Statement

The animal study was reviewed and approved by University of Florida Institutional Animal Care and Use Committee.

## Author Contributions

RP and ME-C performed the experiments and analyzed the data. Statistical analyses were performed by RP. RP and GM interpreted the results of the experiments. RP prepared the figures and drafted the manuscript. ME-C, EG-R, and GM revised the manuscript for intellectual content. RP, ME-C, EG-R, and GM approved the final version of the manuscript. All authors contributed to the article and approved the submitted version.

## Conflict of Interest

The authors declare that the research was conducted in the absence of any commercial or financial relationships that could be construed as a potential conflict of interest.
